# Unveiling the potential of cellulose nanofibre based nitrogen fertilizer and its transformative effect on *Vigna radiata* (Mung Bean): nanofibre for sustainable agriculture

**DOI:** 10.3389/fpls.2024.1336884

**Published:** 2024-01-31

**Authors:** Neha Sharma, Mandira Kochar, Benjamin James Allardyce, Rangam Rajkhowa, Ruchi Agrawal

**Affiliations:** ^1^ The Energy and Resources Institute (TERI) Deakin Nanobiotechnology Centre, Sustainable Agriculture Programme, TERI Gram, Gwal Pahari, Gurugram, India; ^2^ Deakin University, Institute for Frontier Materials, Geelong, Australia

**Keywords:** ammonium chloride-fertilizer, CNF, controlled-release fertilizer, *Vigna radiata*, plant growth, soil nutrient

## Abstract

**Introduction:**

Fertilizer management is crucial to maintaining a balance between environmental health, plant health, and total crop yield. Farmers are overutilizing fertilizers with a mind set to enhance the productive capacity of the field, which adversely impacts soil fertility and causes serious environmental hazards. To mitigate the issues of over-utilization of fertilizers, controlled-release fertilizers were developed using nitrogen fertilizer (ammonium chloride) loaded on cellulose nanofibres (named CNF*N).

**Methodology:**

In this study, the effects of CNF*N were compared with commercial nitrogen fertilizer (ammonium chloride) on *Vigna radiata* (Mung) under greenhouse conditions. The pot experiment was conducted using six treatments: first treatment was control, where the plant was cultivated (T1); second treatment was T2, where the plant was cultivated with CNF to determine the impact of CNF on the plant; third was T3 where commercial ammonium chloride (24 mg/ 2 kg soil) was added to the plant; fourth was T4, where the plant was loaded with CNF, viz. CNF*N contains 4.8 mg of nitrogen; fifth was T5 CNF*N pellet contains 12 mg of nitrogen, and the last sixth treatment (T6) where CNF*N pellet containing 24 mg of nitrogen.

**Results:**

It indicated that the growth parameters were best achieved in T6 treatment. Plant height was at its maximum in the T6 treatment (44.4 ±0.1cm) after the second harvest, whereas the minimum plant height was observed in T1, which was 39.1 ±0.1 cm. Root-to-shoot weight ratio was also maximum in T6 (0.183± 0.02) and minimum in T1 (0.07± 0.01) after second harvesting. The significant difference among the treatments was determined with Tukey’s honestly significant difference (HSD). The nitrogen content (available and total) was significantly higher in the T4, T5, and T6 treatments (0.22, 0.25, and 0.28%) as compared to the control treatments (T1 (0.12%), T2 (0.13%), and T3 (0.14%) during the second harvesting stage (90 days), as nitrogen plays a crucial role in the development of vegetative growth in *Vigna radiata*. The rate of controlled-release nitrogen-fertilizer was found to be optimal in terms of plant growth and soil nutrients; hence, it could potentially play a crucial role in improving soil health and the yield of the crop.

## Introduction

Agriculture is the principal industry responsible for the economic growth and the primary means of subsistence in rural regions of the developing countries like India. Further, to fulfil the food demand of ever-increasing population and to enhance crop yield, excessive fertilizers are utilized, even though a major portion of these fertilizers are lost to the environment through hydrolysis, photolysis, or volatilization (Shojaei et al., 2019). To mitigate the issues of leakage of fertilizer, controlled release fertilizers were developed that allow the control release of fertilizer on the targeted site through diffusion process. Controlled release fertilizer based on CNF was used on Mung bean (*Vigna radiata* L. Wilczek). *Vigna radiata* is one of the crops drastically affected due to inadequate and unbalanced fertilizer (Jain et al., 2007; Asaduzzaman et al., 2008). It is widely known as green gram or bean, belongs to the legume family Phaseoleae and is presumed to be a native crop of India (Ikraam, 2002; Hua, 2006; Hussain et al., 2011; Alemu, 2016; Cheng, 2016). When soil nitrogen levels are low, a modest amount of nitrogen fertilizer is applied to encourage rhizobia development and boost the growth of vigorous *Vigna radiata* seedlings (Yayeh et al., 2017). *Vigna radiata* cannot properly fix atmospheric nitrogen during the early development phases before the branches emerge because it has few or no rhizobia. Nitrogen is one of the key components of amino acids and proteins; phosphorous promotes root development and plays a key role in metabolic processes, while potassium is important for physiological processes like osmoregulation, assimilate transport, and enzyme activation (Yanni et al., 2001). Increased nitrogen fertilizer treatment during the early growing stage encourages vegetative growth and fosters circumstances that are favourable for high yield. Rhizobia proliferate, and the plant’s capacity to fix atmospheric nitrogen rises; however, during the late development stage, if too much nitrogen fertilizer is used, rhizobia activity gets limited. And the development of the flower bud and the subsequent yields are hampered in such circumstances (Sadeghipour et al., 2010). Additionally, the quality of agricultural produce and the microbiota of the soil have been found to be impacted along with the nutrient holding capacities, nutrient usage efficiency, soil fertility degradation, and increased soil-borne diseases (Naeem et al., 2006).

Therefore, a balanced fertilizer dose application and careful manure management can increase *Vigna radiata* production and quality (Krishnamoorthy and Rajiv, 2017). Sustainable agriculture solutions to improve the bioavailability of nutrients to plants entail increasing a farmer’s net gains from agricultural output. This may be accomplished by utilizing biopolymers-based controlled-release fertilizers (Nair et al., 2019). This study involves the cellulose nanofibre (CNF)-based nitrogen-fertilizer, in which CNF works as a carrier and delivers the fertilizer in a controlled manner. The purpose of this study is to guide the crop production of *Vigna radiata* using CNF*N as a controlled-release fertilizer system with the aim of overcoming the limitations of traditional fertilizers and assisting effective *Vigna radiata* cultivation. For this, the greenhouse experiment was performed, where six different treatments in 12 replicates were given and their plant physiological parameters (plant length, leaf area, root-to-shoot ratio, primary branches, pH, electrical conductivity, organic carbon, nitrogen analysis, etc.) were determined.

## Materials and methods

### Materials

Rice straw was collected from the district of Mathura, Uttar Pradesh. The cellulose nanofibres derived from rice straw were obtained through TEMPO-oxidation, as described in our previous study (Sharma et al., 2023b). Fertilizer was procured from the chemical lab at TERI. *Vigna radiata* seeds (Morya variety) were purchased from the local market in Gurgaon, India. The soil used in the experiment was collected from a farm located in TERI Gram, Haryana, India (28.4275° N, 77.1465° E) from a depth of 0-20 cm.

### Soil characterization

Soil was sieved and autoclaved three times to eliminate unwanted contaminants was and was characterized for physicochemical parameters such as pH using pH meter, electrical conductivity by conductivity meter, organic carbon, total and available nitrogen by Kjeldalh method as mentioned in our previous study (Sharma et al., 2023c) (**Table 1**).

**Table 1:** Physicochemical properties of the soil.

Parameters	Values
pH	7.2 ± 0.005
Electrical Conductivity (µS/cm)	140.0 ± 0.05
Organic carbon (%)	0.0011 ± 0.0005
Available N (%)	0
Total N (%)	0

### Experimental site

Greenhouse pot trials were performed at Polyhouse 3 of the TERI-Deakin Nanobiotechnology Centre, TERI-Gram, Gurugram, India (28.4275° N, 77.1465° E) (**Supplementary Figure S1**). The study was conducted for three months, beginning in the fourth week of March 2023 and continuing until the fourth week of June 2023 maintained with temperature of day/night (27/25°C). As the region had the climate characteristics of plains, irrigation was performed on a daily basis until the end of the study.

### Pot studies on *Vigna radiata* under greenhouse conditions

The Morya variety of *Vigna radiata* is high-yielding and has a high tolerance to powdery mildew diseases caused by *Podosphaera fusca* and *Erysiphe polygoni* (Yin et al., 2018). The greenhouse study was conducted following the method of Yin et al. with slight modifications (He et al., 2020). The soil was autoclaved three times and added to sterilized 2-kg pots. Prior to the pot study, the *Vigna radiata* seeds of the cultivar were investigated for their viability. The seeds were washed with Tween 20 detergent for 3 min followed by 3 to 4 washings with water. Further, surface sterilization was performed with 0.01% HgCl_2_ for 1 min followed by five washings with autoclaved water. The surface-sterilized seeds were placed in the petri dish, and their germination was checked. After seed germination, one seed per pot was sown and evaluated further (**Supplementary Figure S2**).

The pot experiments using controlled-release nitrogen-based fertilizer were conducted with six different treatments, and their details are mentioned in **Table 2** with the aim to determine the overall growth of *Vigna radiata*. All treatments were set up in 12 replicates (72 trial pots) for two harvestings. In the first harvesting stage, 36 pots were harvested after 45 days of plantation when the trifoliolates were developed, and the second harvesting stage was performed on the 90^th^ day of plantation when the pods with sufficient seeds were developed.

**Table 2:** Codes for the different nitrogen-based fertilizer treatments.

Treatments	Description
t 1	Untreated Plant- Negative Control 1
t 2	Plant+ CNF- Negative Control 2
t 3	Plant +Ammonium chloride (24 mg)- Positive control
t 4	Plant + CNF*N- 4.8 mg nitrogen
t 5	Plant + CNF*N- 12 mg nitrogen
t 6	Plant + CNF*N- 24 mg nitrogen

### Plant harvesting and soil nutrient analysis

For harvesting, the whole plant was carefully uprooted from the soil, retaining the fine roots. This was followed by the washing of the roots to remove the adhered soil several times with distilled water. The plant shoot and root were separated and oven-dried for 3 days at 55°C. The shoot and root dry weights were recorded, and the roots-to-shoot ratio was determined. The leaves on the plant were counted; leaf area was calculated, and the number of primary branches was also counted for both the first and second harvesting stages (Sharma et al., 2020). The soil pH and electrical conductivity were analysed using a pH metre and a conductivity metre, respectively. The conductivity metre was calibrated with a standard KCl solution, whereas the pH metre was calibrated using several pH buffers (4.0, 7.0, and 9.0) (Potdar et al., 2021).

For nitrogen estimation in soil, the Kjeldahl method was used, and the distillation process was employed, where 20 g of soil, 100 ml of potassium permanganate, and 100 ml of sodium hydroxide were added into a digestion tube. The released ammonia was collected in boric acid using a distillation unit, where it forms ammonium borate and gets collected in a conical flask. The ammonium borate solution was titrated with sulfuric acid (0.1N H_2_SO_4_) till the color changed from pink or red to green (Lelago et al., 2016). The percentage of nitrogen was calculated using Equation 1:


(1) %N=1.4V×N÷wtof sample

Where, V is acid used in the titration (ml), and N is the normality of the standard acid.

### Statistical analysis

All plant growth data and soil data were analysed by one-way analysis of variance (ANOVA) with the factors in the treatment analysis. The significant difference among the treatments was determined with Tukey’s honestly significant difference (HSD) using OriginPro 8.5.

## Results

### Physiochemical properties of soil

The soil physico-chemical parameters were checked before the initiation of the pot studies. The results obtained are tabulated in **Table 1**. The soil pH was analyzed to be 7.24, which was moderately neutral. According to the South African Department of Agriculture, Forestry, and Fisheries, the best production of *Vigna radiata* requires sandy loam soil with good drainage at a pH of 6.3–7.2. Neutral soil with a pH of 6.7–7.3 and silty clay soil texture might have fewer drainage problems for *Vigna radiata*. The electrical conductivity (EC) of the soil was monitored to be low (140.0 ± 0.05 µS/cm) indicating the soil was free of salts. Organic carbon and nitrogen contents were also depicted in the table below. The soil was deficient in nitrogen and hence appropriate for studying the nitrogenous fertilizers effect (Mota et al., 2021).

### Impact of CNF*N controlled-release nitrogen fertilizer on the growth of *Vigna radiata*

#### Plant height of the *Vigna radiata* plant

**Figure 1:**
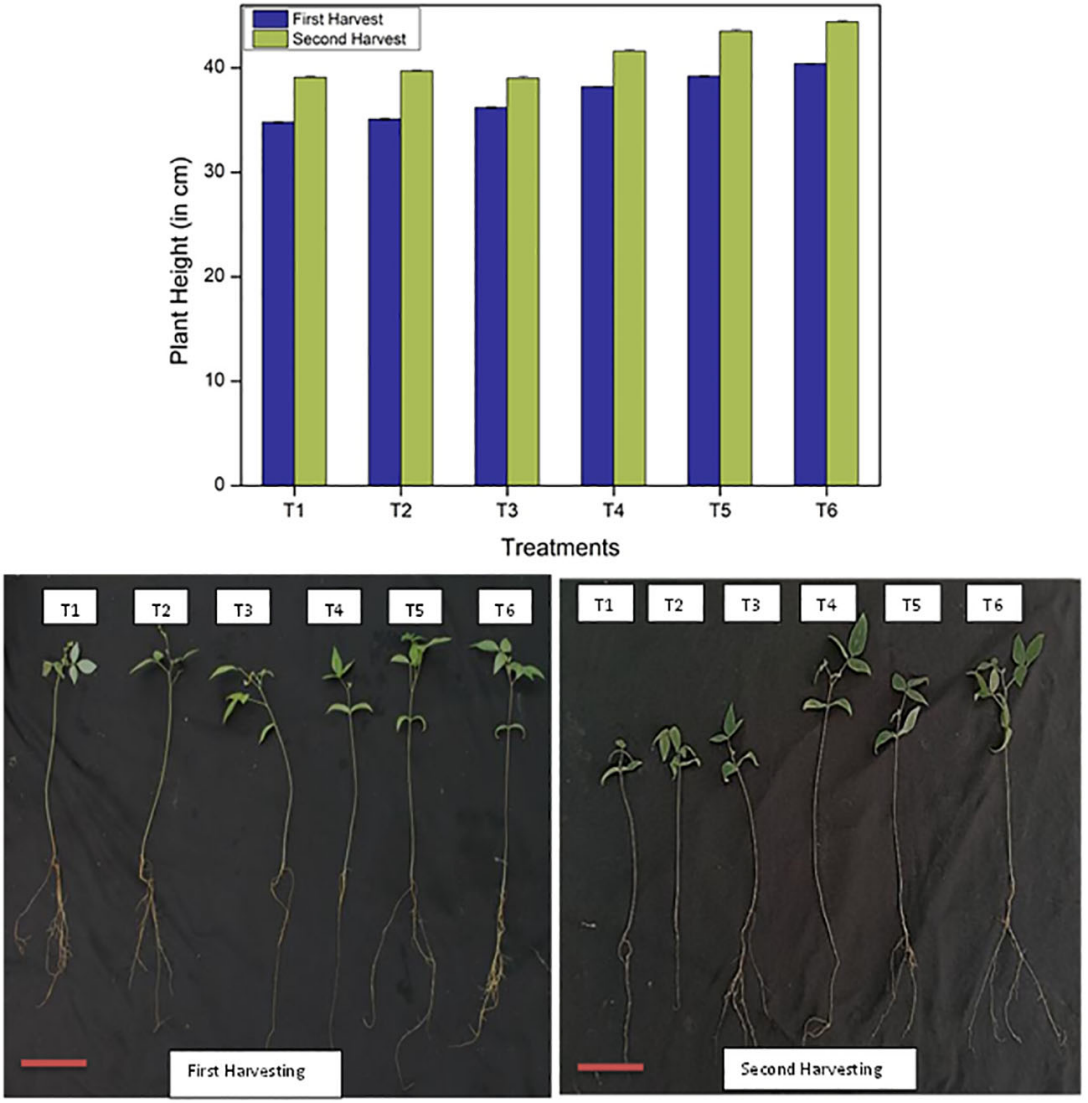
Image of the plants after the first (45 days) and second (90 days) harvesting stages showing the effect of six different treatments on the phenotype and height of the plant All the measurements were done in replicates of six plants (bars represent standard deviation; scale bar = 1 cm).

Plant height, as a principal agronomic character, is the length of the *Vigna radiata* plant and is closely related to the yield of the crops. Plant height increased with the increase in CNF*N, as checked after both the first (after 45 days) and second (after 90 days) harvesting. The maximum height of the plant was observed in treatment 6 (T6), where 50 mg of CNF*N was added, as compared to control treatment 1 (T1), where no fertilizer was added. After first harvesting, the height in T6 was 40.4 ± 0.04 cm as compared to 34.8cm ± 0.08 in T1. T2 treatment showed 35.1 ± 0.09, 36.2 ± 0.1 in T3, 38.2 ± 0.05 in T4, and 39.2 ± 0.08 in T5. After second harvesting, the height in T6 was 44.4 ± 0.1 cm while the height in control (T1) was 39.1 ± 0.1 cm (**Figure 1**). Other treatments indicated 39.1 ± 0.1 in T2, 39.0 ± 0.2 in T3, 41.6 ± 0.1 in T4, and 43.5 ± 0.1 in T5. Thus, the increase in plant height is probably due to increased nitrogen content until the 90th day, as T6 treatments represent controlled-release fertilizer which led to enhanced root development and crop growth. All the measurements were the average of six replicates of each treatment.

#### Dry weight of root and shoot of *Vigna radiata*

The dry weight of plants tends to provide a precise assessment of plant biomass by removing changes caused by water content. Plant performance in response to photosynthetic ability, nutrition, and environmental factors can be directly correlated with plant total biomass. The details of the root and shoot dry weights of plants are represented in **Table 3**.

**Table 3:** Dry weight of root and shoot in the first and second harvesting stages.

Treatments	Root weight (in g)	Shoot weight (in g)
First Harvesting		
T1	0.013 ± 0.002	0.39 ± 0.04
T2	0.012 ± 0.005	0.27 ± 0.06
T3	0.014 ± 0.009	0.36 ± 0.07
T4	0.018 ± 0.007	0.33 ± 0.06
T5	0.017 ± 0.006	0.28 ± 0.02
T6	0.029 ± 0.005	0.3 ± 0.04
Second Harvesting		
T1	0.07 ± 0.001	0.9 ± 0.02
T2	0.07 ± 0.002	0.82 ± 0.02
T3	0.07 ± 0.001	0.87 ± 0.04
T4	0.08 ± 0.002	0.73 ± 0.02
T5	0.1 ± 0.001	0.9 ± 0.02
T6	0.2 ± 0.001	1.09 ± 0.02

#### Root-to-shoot ratio of *Vigna radiata*

**Figure 2:**
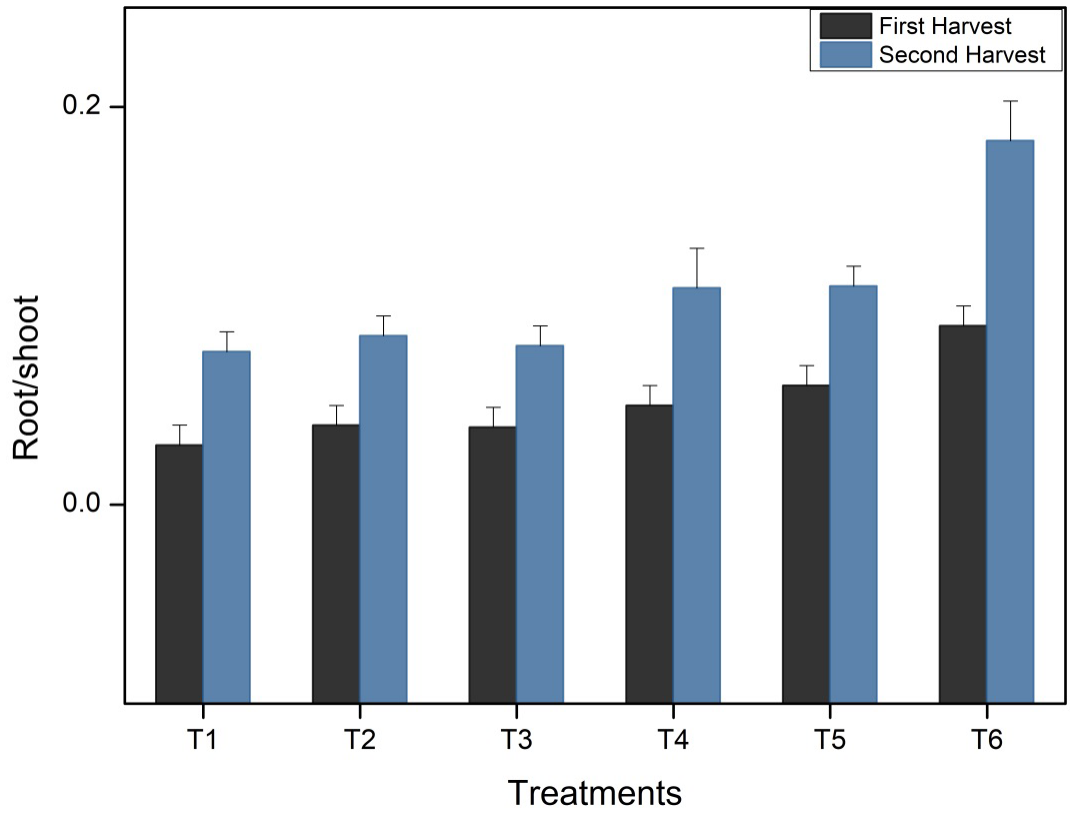
Root-to-shoot ratio of *Vigna radiata* plants at first and second harvesting stage (Bars representing standard deviation).

The ability of a plant to absorb water and nutrients from its surroundings is dependent on its roots. A healthy root system is essential to the overall health of the plant. The root-to-shoot ratio is an important parameter to monitor plant health. The root-to-shoot ratio was highest for T6 treatment during both the first (0.09± 0.01) and second harvestings (0.183± 0.02) as compared to the control T1 (without any fertilizer) with the lowest root-to-shoot ratio during the first (0.03 ± 0.01) and second (0.07± 0.01) harvestings. Other treatments T2 showed 0.04± 0.01, 0.039± 0.01 in T3, 0.05± 0.01 in T4, and 0.06± 0.01 in T5 root-to-shoot ratio in first harvesting followed by second harvesting, which showed 0.08± 0.01 in T2, 0.08± 0.01 in T3, 0.10± 0.02 in T4, and 0.11± 0.01 in T5 as represented in **Figure 2**.

**Figure 3:**
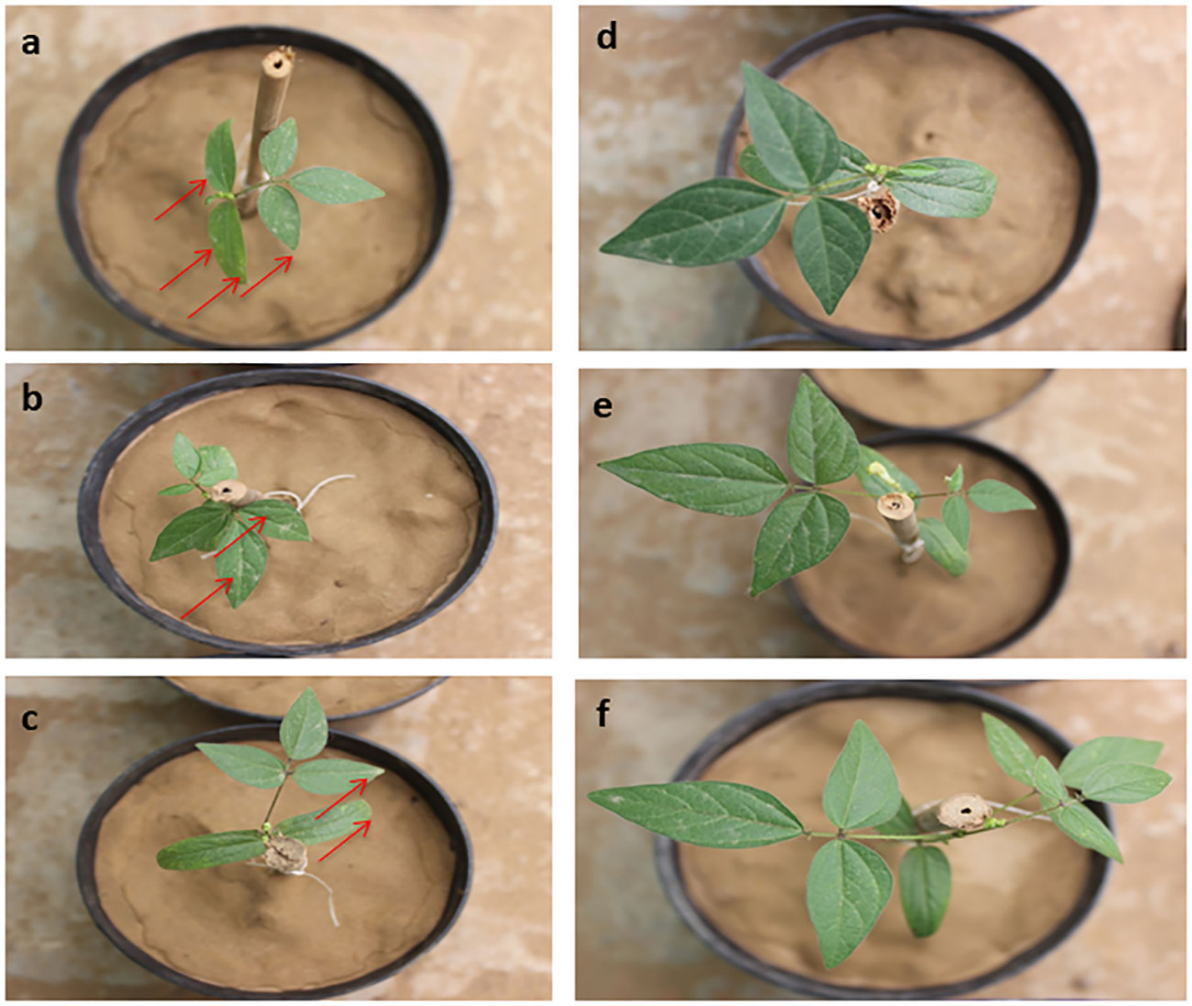
Photographs of the *Vigna radiata* with different soil treatments at the second harvesting stage or after 90 days of growing phase: **(A)** T1, **(B)** T2, **(C)** T3, **(D)** T4, **(E)** T5, and **(F)** T6, where the red arrow represents the yellow spots on the leaf due to nitrogen deficiency.

**Figure 2** represents the results of the dry weight of the root-to-shoot ratio of *Vigna radiata* with six different treatments (T1-T6) after 45 and 90 days. The outcomes of the study revealed that treatment T6 showed significantly better results as compared to the other treatments, and the plants harvested had better vigour and health (**Figure 3**).

#### Assessment of the number of leaves

**Figure 4:**
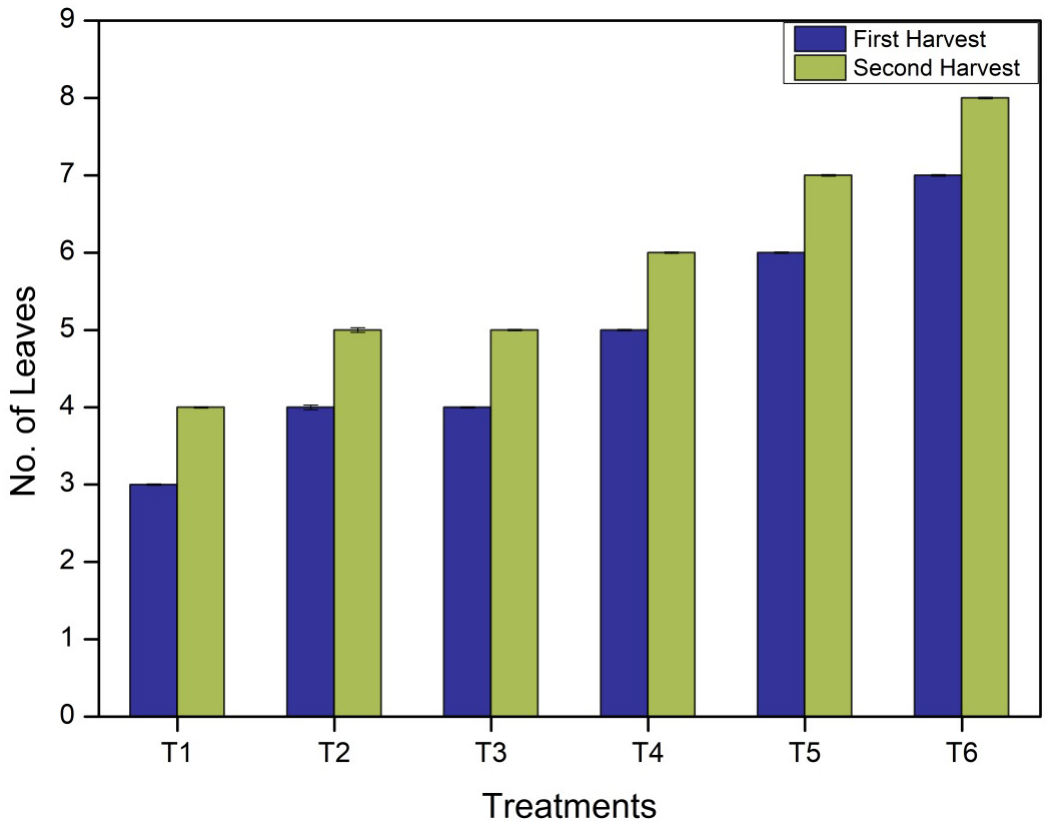
Number of leaves at the first and second harvesting stages of *Vigna radiata* plants (bars represent the standard deviation).

The number of leaves per plant is significantly impacted by the time of the fertilizer application and the type of the fertilizer. The highest number of leaves (7 in the first harvesting stage and 8 in the second harvesting stage) were found in the T6 treatment where 50 mg of CNF*N was added to the soil. In control T1 (without any fertilizer), 3 leaves were found after the first harvesting stage, and 4 leaves were obtained after second harvesting stage. Other treatments, such as T2, showed 4, 4 in T3, 5 in T4, and 6 leaves in the T5 in first harvesting, followed by the second harvesting, which showed 5 in T2, 5 in T3, 6 in T4, and 7 leaves in T5, as represented in **Figure 4**.

#### Leaf area of the *Vigna radiata* plant

**Figure 5:**
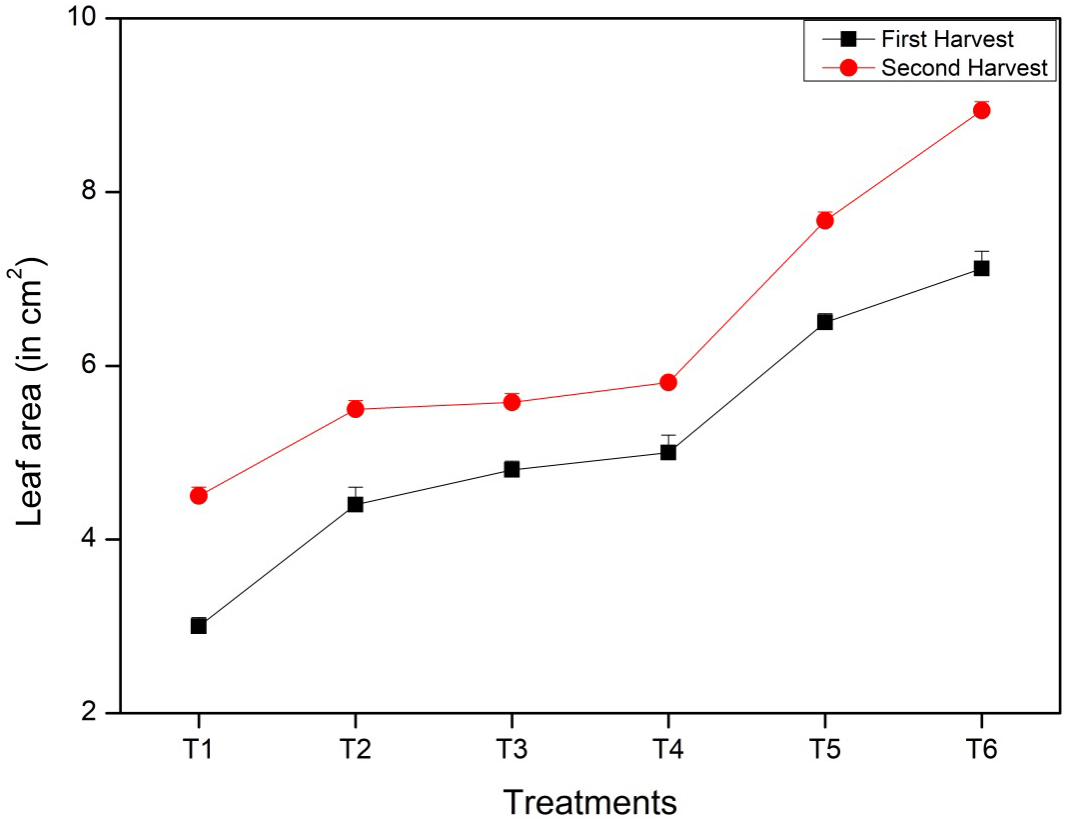
Leaf area at the first and second harvesting stages of *Vigna radiata* plants.

Similar to the number of leaves per plant, the leaf area was also significantly affected by the nitrogen rates. The plant with the maximum leaf area may also have higher photosynthesis because of increased nutrition during the growth stages. The maximum leaf area was recorded in the T6 treatment i.e., 7.12 cm^2^ in the first harvesting stage and 8.94 cm^2^ in the second harvesting stage, whereas the minimum leaf area (3 cm^2^ in the first harvesting stage and 4.5 cm^2^ in the second harvesting stage) was obtained in the control (T1) with no fertilizer applied. Other treatments showed leaf areas of 4.4 cm^2^ in T2, 4.8 cm^2^ in T3, 5 cm^2^ in T4, and 6.5 cm^2^ in T5 in the first harvesting, followed by the second harvesting, which showed 5.5 cm^2^ in T2, 5.5 cm^2^ in T3, 5.8 cm^2^ in T4, and 7.6 cm^2^ in T5 (**Figure 5**). Thus, the mean leaf area was observed to increase with the controlled-release fertilizer using CNF*N. Interestingly, there was only a marginal difference among the control T1 and T3 (bulk ammonium chloride), whereas no significant differences in parameters between the T2 (cellulose nanofibre pellet), T3 (bulk ammonium chloride), and T4 (CNF*N, 10 mg) were observed. A significant improvement was obtained with increasing the dosage of the CNF*N from 10 mg to 50 mg. An exponential increase was observed in the leaf area, i.e., from ~3 to ~7.1 cm^2^ in the first harvest and ~ 4.5 to~ 8.9 cm^2^ in the second harvest.

#### Number of primary branches

The results indicated that the number of primary branches was significantly affected by the addition of N-fertilizer. Notably, the highest numbers of primary branches (4 in the first harvesting stage and 7 in the second harvesting stage) were recorded in T6 treatment as compared to the control (T1) (2 in first harvesting stage and 4 in second harvesting stage), where no fertilizer was applied. Other treatments showed primary branches of 4.4 cm^2^ in T2, 4.8 cm^2^ in T3, 5 cm^2^ in T4, and 6.5 cm^2^ in T5 in first harvesting, followed by second harvesting, which showed 5.5 cm^2^ in T2, 5.5 cm^2^ in T3, 5.8 cm^2^ in T4, and 7.6 cm^2^ in T5. This implied that higher vegetative growth was achieved when there was a higher availability of nutrients due to the controlled delivery of the fertilizer via a CNF-based N-fertilizer formulation.

### Physiochemical parameters of soil at the first and second harvesting stages of the mung bean plant

The soil nutrient contents were monitored during the two post-harvest stages (45 days and 90 days) of the mung bean cultivation, as shown in **Table 4**. No significant difference was observed in the soil pH and organic carbon content among the controls (T1, T2) and the treatments (T3-T6) that were given to the mung plant [34]. In this study, the slight basic pH, which causes the continual removal of NH_4_^+^ ions from the nitrogen-fertilizer loaded CNF and neutralizes the hydronium ions (OH^-^) in the soil, favors the equilibrium shift, making the process self-propagating. Further, the CNF in the later stage will be depolymerized by microbes into oligomers or dimers, which serve as carbon sources for the plant and further contribute to plant yield (Zhou et al., 2011).

**Table 4:** Physico-chemical parameters of soil after the first and second harvesting stages of *Vigna radiata*.

Treatment	pH	Organic carbon (%)	Electrical conductivity (µS/cm)
First Harvesting Stage (45 days)			
T1	8.3 ± 0.01	0.0023 ± 0.02	145.9 ± 0.2
T2	8.8 ± 0.05	0.0021 ± 0.01	149.4 ± 0.2
T3	8.3 ± 0.01	0.0023 ± 0.01	208.9 ± 0.1
T4	8.6 ± 0.01	0.002 ± 0.01	164.6 ± 0.1
T5	8.7 ± 0.01	0.002 ± 0.02	170.2 ± 0.1
T6	8.7 ± 0.01	0.002 ± 0.01	175.3 ± 0.2
Second Harvesting Stage (90 days)			
T1	8.4 ± 0.08	0.002 ± 0.001	148 ± 0.1
T2	8.3 ± 0.05	0.002 ± 0.1	152 ± 0.2
T3	8.4 ± 0.01	0.002 ± 0.02	125.0 ± 0.02
T4	8.4 ± 0.01	0.002 ± 0.02	169.9 ± 0.08
T5	8.5 ± 0.01	0.003 ± 0.001	170 ± 0.08
T6	8.4 ± 0.01	0.003 ± 0.001	180 ± 0.02

The electric conductivity was highest in the T3 treatment as compared to the other treatments and ranged from 145 to 175 µS/cm. The maximum EC in treatment 3 (T3) in the first harvesting stage was observed as the ammonium chloride was added to the soil in bulk during the treatment. The electrical conductivity increased with the solubilization of water-soluble ammonium chloride fertilizer which also increased the salt levels in the soil. As a major portion of the ammonium chloride added was already released or leaked until the first harvesting stage, the EC values of the T3 treatment decreased after the first harvesting stage. During the second harvesting stage, the EC was found to be in the range of 125 to 180 µS/cm. The maximum EC was found in T6, owing to the controlled-release of nitrogen-fertilizer loaded onto CNF.

### Estimation of nitrogen in soil

**Figure 6:**
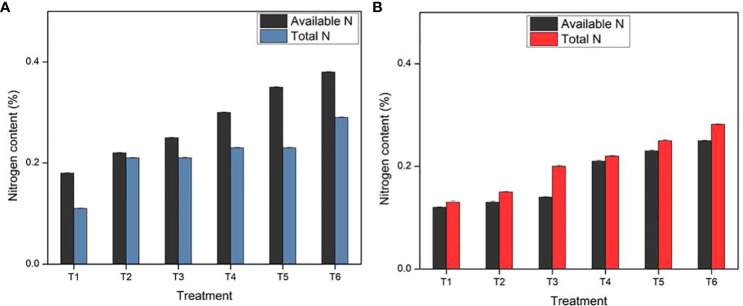
Percentage of nitrogen contents in soil: **(A)** first harvesting stage; **(B)** second harvesting stage of *Vigna radiata* (bars represent the standard deviation).

The nutrient nitrogen (N) is essential for plant growth and the overall yield of the crop. Significantly higher nitrogen content (available and total) was observed in the CNF*N treatments (T4, T5, and T6) as compared with the negative control treatments (T1, T2) and positive control treatment (T3) during the first harvesting stage (45 days). The positive control was bulk fertilizer that was highly soluble in water and released during the initial growing phase, whereas the CNF*N treatments showed controlled release of nitrogen until the 45^th^ day with maximum concentrations. Similarly, in the second harvesting stage, the soil components, mainly nitrogen, were analyzed for all the treatments to determine the nutrient contents in the soil. Nitrogen content was highest in the T6 treatment as compared to the control. The nitrogen content in T3 treatment where commercially available ammonium chloride was added in bulk was less, as ammonium chloride was highly soluble in water, so there are probably more chances of leakage of the salt in free form as compared to the salt loaded on CNF. Similar pattern was observed in the entire greenhouse study, where the maximum soil nutrients, plant height, leaf area, and other growth parameters were found in nitrogen-fertilizer loaded CNF treatments (T4, T5, and T6). Treatments T4, T5, and T6 were observed to represent no deficiency in the soil nutrient (nitrogen), which was attributed to the substantial release of sufficient quantities of nitrogen from the designed CNF*N formulation even after 90 days of the second harvesting. This observation was attributed to the controlled-release of nitrogen-fertilizer loaded onto the CNF, which regulates and improves the nutrient uptake efficiency of the plant via the controlled release of nutrient, as shown in **Figure 6**.

The results were further corroborated by the yellowing of leaves in treatments T1, T2, and T3 after 90 days of the growing phase (**Figures 3A–F**) which indicated the lesser availability of nitrogen in the T3 treatment, where commercial ammonium chloride added in bulk was prone to microbial immobilization or leaching losses, whereas the T1 and T2 treatments were without any fertilizer, one propagating with only on the original soil and the other on soil with unloaded CNF. However, the treatments T4-T6 with controlled-release N-fertilizer loaded CNF at nitrogen loadings comparable to T3 did not show such manifestations anytime during the 90 days growth period.

Thus, the formulated CNF*N (nitrogen-fertilizer loaded CNF) appeared as a controlled-release fertilizer system that maintained the nitrogen content even after 90 days of harvesting. The results indicate that the nitrogen content in the soil was high in the replicates treated with the formulation, and hence the designed formulation could be utilized as an alternative to the commercial fertilizer, which had to be applied in bulk and was hence prone to volatization and leaching, leading to poor adsorption by the plants.

## Discussions

The controlled release fertilizers are the smartest tools for sustainable agriculture. However, the impact of controlled release fertilizer on crops must be carefully evaluated before field implementation (Sharma et al., 2023a). The physiochemical parameter of soil plays critical role for plant growth (Yin et al., 2018). Numerous studies showed fertilizer impact on *Vigna radiate.* A report by Mota et al. showed the dosage of NPS (150 kg) per hectare on *Vigna radiata*, where the Shewa Robit variety showed maximum growth of the plant around 67.75 cm, whereas the minimum height of 59.0 cm was obtained in plants without additional fertilizer. Therefore, the causes of the maximum plant height were the genetic differences between varieties and enhanced NPS fertilizer. It could also be the result of a climate that is favourable up to physiological maturity, particularly when it comes to timely rainfall throughout the growth season (Khandare et al., 2020). According to another report, nitrogen plays a critical role in stimulating vegetative growth, thus impacting the overall yield of the plant (Chen et al., 2018).

A report by Khandare et al. observed plant dry matter of about 10.8 and 10.5 g/plant, both with 75% NP + liquid inoculation (*Azotobacter*-PSB; 625 mL/ha) and 75% NP + carrier inoculation (*Azotobacter*-PSB; 10 kg/ha), respectively (Alori et al., 2017), which indicated the carrier was beneficial to the plant. A report by Chen et al., showed that a moderate rate of nitrogen-fertilization plays a crucial role in root elongation, increasing surface area and volume in soil. The entire length of the root was found to grow to 25 cm using the minirhizotron method (Houmani et al., 2015).

The plant roots also play a critical role in maintaining the rhizosphere environment, as root exudate releases organic acids and protons (H^+^). The rhizosphere in the soil plays an essential role in facilitating the interaction between plant roots and microbes, which further enhances the mineralization of nitrogen and further enhances the plant’s net nitrogen assimilation (Dong et al., 2008; Karuku, 2019; Sharma et al., 2023c). Multiple studies have shown that the overall chlorophyll content in the leaves increased with excessive application of nitrogen-fertilizer but it may impact the carbon and nitrogen metabolism within the soil. Thus, under high nitrogen-fertilizer concentration the root growth was impacted, but the leaf numbers were increased (Kawte et al., 2020; Wang et al., 2020). An increased number of branches of plant indicated improved growth, which is the result of sufficient availability of nutrients at growth stages leading to efficient photosynthetic activity (Zhou et al., 2011). A similar study was conducted by Fernandes et al., where the water-soluble fertilizer showed a maximum EC of approximately 3 dS/m (Fernandes de Oliveira Braghin et al., 2019).

Thus, the utilization of CNF-based controlled-release fertilizer has proven to be highly effective in promoting the growth and development of *Vigna radiata* plants. The controlled release mechanism ensures a sustained and balanced supply of nutrients, optimizing nutrient uptake by the plants. This approach not only enhances the overall yield of *Vigna radiata* but also contributes to resource efficiency and environmental sustainability. The results suggest that CNF-based controlled release fertilizers can be a promising strategy for improving crop productivity while minimizing the environmental impact associated with traditional fertilization methods.

## Conclusion

The performance of CNF*N controlled-release fertilizer has not only slowed down the nutrient release to the soil but also improved the soil fertility profile with the polymerization of CNF that will be utilized by microbes. The results of the study showed that plant height was highest in T6 treatment (44.4 ± 0.1 cm), whereas the less plant height was observed in T1, which was 39.1 ± 0.1 cm, after second harvesting, and the root-to-shoot ratio was maximum in T6 (0.183± 0.02) and minimum in T1 (0.07± 0.01) after second harvesting. This suggested the controlled-release of nitrogen-fertilizers tuned in accordance with the plant’s requirement for nitrogen. Thus, a CNF-based controlled fertilizer system could be adopted as an eco-friendly and biodegradable alternative to the practice of commercial application of the fertilizers by broadcasting, which otherwise leads to nutrients leaching to the water bodies, limited absorption by plants, and thus causing eutrophication and serious health hazards.

## Data availability statement

The original contributions presented in the study are included in the article/**Supplementary Material**. Further inquiries can be directed to the corresponding author.

## Author contributions

NS: Conceptualization, Data curation, Formal analysis, Investigation, Methodology, Writing – original draft, Writing – review & editing. MK: Project administration, Resources, Supervision, Validation, Visualization, Writing – review & editing. BA: Supervision, Validation, Writing – review & editing. RR: Project administration, Supervision, Validation, Writing – review & editing. RA: Project administration, Supervision, Validation, Visualization, Writing – review & editing.
