# Supplementary material for: Unveiling the potential of cellulose nanofibre based nitrogen fertilizer and its transformative effect on Vigna radiata (Mung Bean): nanofibre for sustainable agriculture
Source: Front Plant Sci. 2024 Jan 31;15:1336884. doi: 10.3389/fpls.2024.1336884 (PMC10864528; doi:10.3389/fpls.2024.1336884)
Supplement: Supplementary file 1 [file DataSheet_1.docx]

**Supplementary**

**Cellulose nanofibre based controlled release nitrogen fertilizer and its impact on *Vigna radiata* (Mung Bean)**

Neha Sharma^1, 2^, Mandira Kochar^1^, Benjamin James Allardyce^2^, Rangam Rajkhowa^2^, Ruchi Agrawal^1*^

^1^TERI Deakin Nanobiotechnology Centre, Sustainable Agriculture Programme, TERI Gram, Gwal Pahari, Gurugram, India

^2^Deakin University, Institute for Frontier Materials, Geelong, Australia

^*^Corresponding author.

E-mail: [ruchi.agrawal@teri.res.in](mailto:ruchi.agrawal@teri.res.in)

***Experimental site****-* The experimental site is located within the campus (Teri Gram) on Gurgaon-Faridabad road.

*
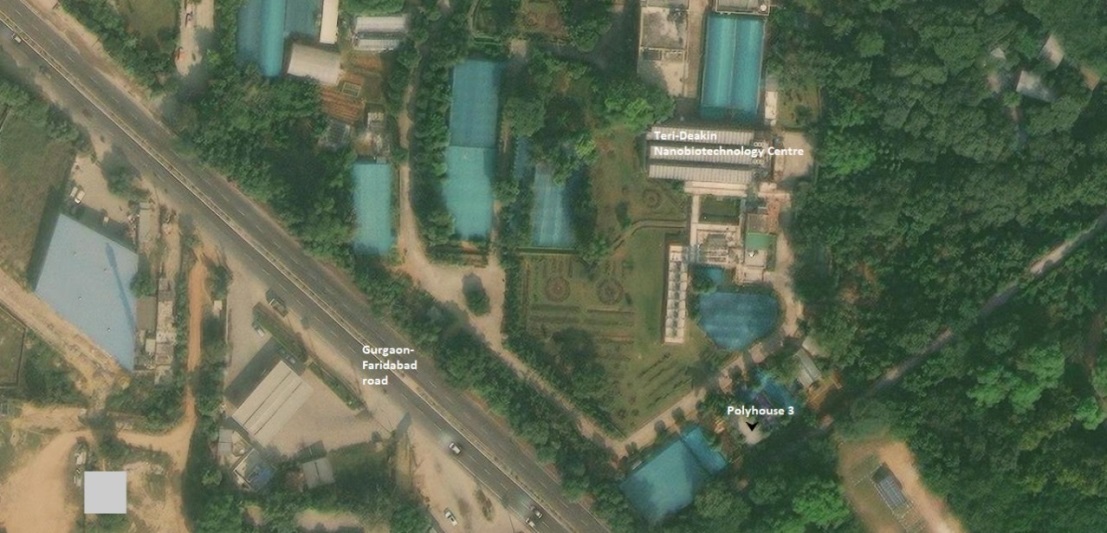
*

**Fig.S1.** Aerial view of experimental site named polyhouse 3

***Pot study experimental plan-*** The pot study was performed with six treatments and there were 12 replicates of each treatment. Total of 72 pots were involved in the study. In first harvesting 36 pots (six from each treatment) was withdrawn for estimation of nutrient and plant parameters.


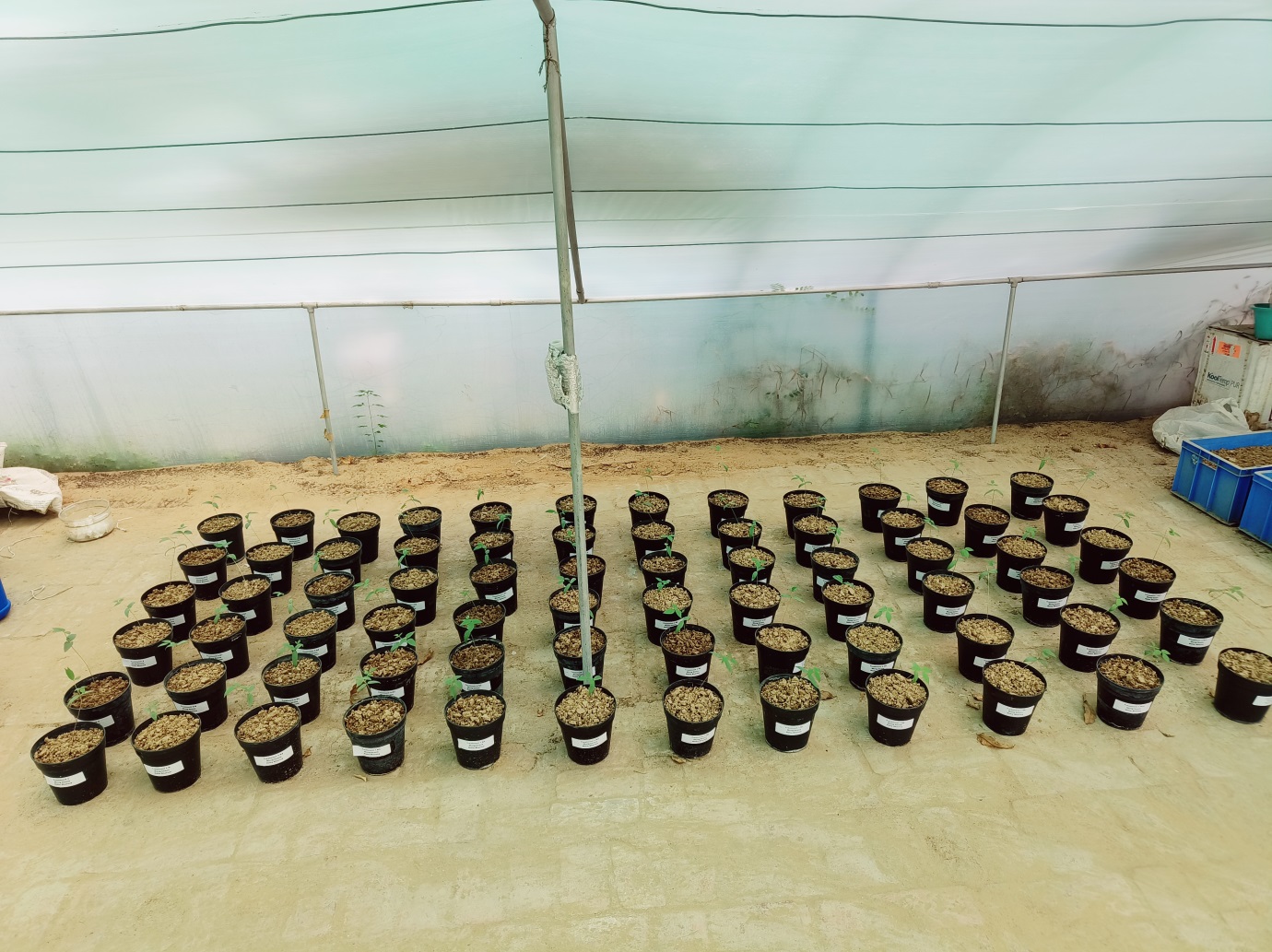


T6

T5

T4

T3

T2

T1


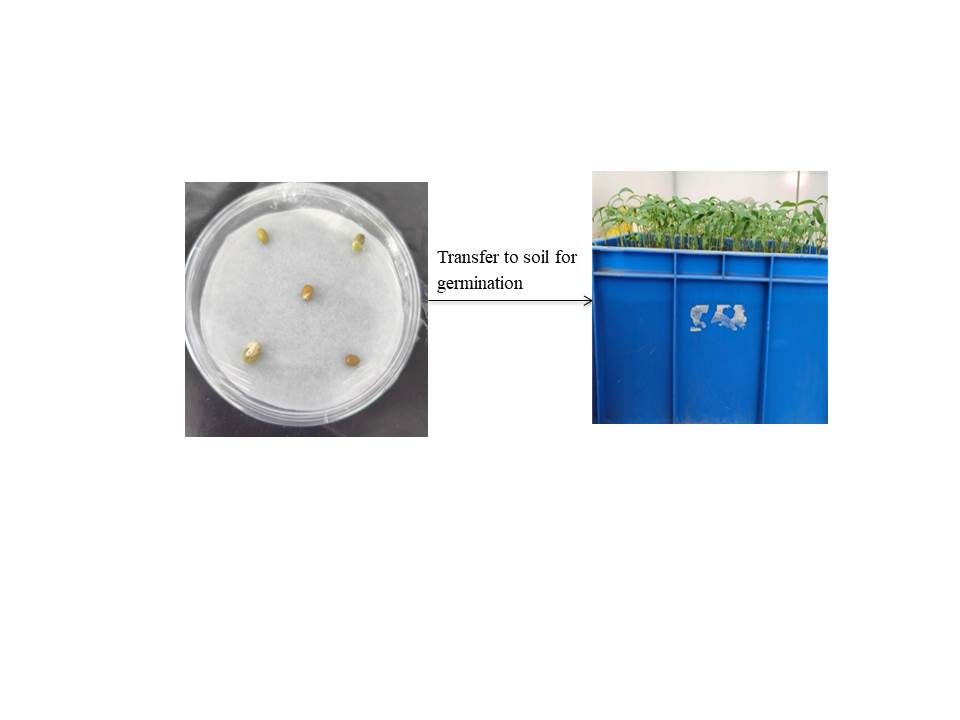


**Fig.S2.** Representation of seed germination and transferring into the pots treated with six different treatments (T1-T6)

***Statistical analysis:*** One-way ANOVA using Turkey method for plant length after treatment


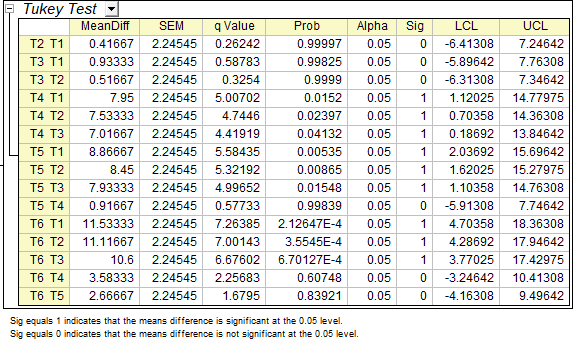

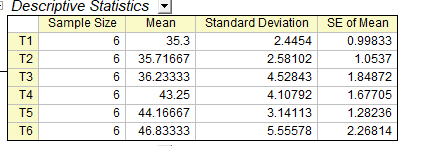

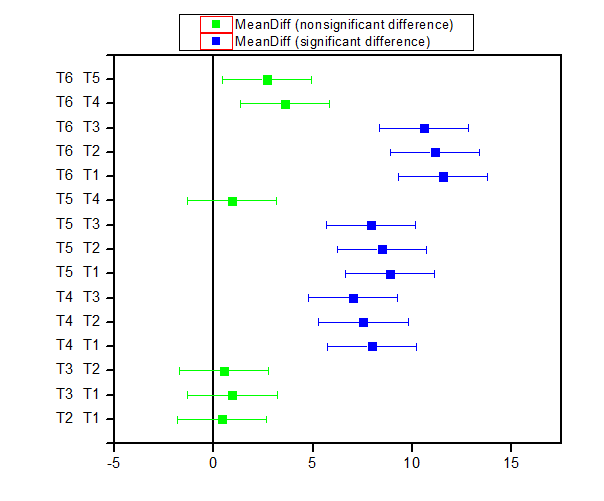


The result reflects the controlled-release fertilizer T4, T5 and T6 were significantly different from the control and commercial ammonium chloride. Thus, the study corroborate that the controlled release treatment release the nutrient in a control-manner that improves the growth of plant.
